# Supplementary material for: VR Realism Scale—Revalidation of contemporary VR headsets on a Polish sample
Source: PLoS One. 2021 Dec 21;16(12):e0261507. doi: 10.1371/journal.pone.0261507 (PMC8691612; doi:10.1371/journal.pone.0261507)
Supplement: S1 Appendix — (PDF) [file pone.0261507.s001.pdf]

Kwestionariusz realizmu wirtualnej rzeczywistości

Dziękujemy za zainteresowanie naszym badaniem. Wypełnienie kwestionariusza zajmie kilka minut. Odpowiedz na pytania odnosząc się do Twojego doświadczenia podczas korzystania z symulatora. Nie ma tutaj odpowiedzi dobrych i złych, liczy się tylko Twoja opinia. Odpowiadaj korzystając z poniższej skali:

Całkowicie się <u>nie</u> zgadzam	Raczej się <u>nie</u> zgadzam	Pół na pół	Raczej się zgadzam	W pełni się zgadzam
-2	-1	0	1	2

**Refleksy w przestrzeni wirtualnej wydawały się naturalne.**	-2 -1 0 +1 +2
**Światło i cienie w przestrzeni wirtualnej były realistyczne.**	-2 -1 0 +1 +2
**Przestrzeń wirtualna sprawiała wrażenie trójwymiarowej.**	-2 -1 0 +1 +2
**Kolorystyka przestrzeni wirtualnej sprawiała naturalne wrażenie.**	-2 -1 0 +1 +2
**Proporcje w przestrzeni wirtualnej były realistyczne.**	-2 -1 0 +1 +2
**Sylwetki wirtualnych osób były naturalne.**	-2 -1 0 +1 +2
**Gestykulacja wirtualnych osób była naturalna.**	-2 -1 0 +1 +2
**Zachowanie wirtualnych osób w przedstawionej scenie było autentyczne.**	-2 -1 0 +1 +2
**Mimika wirtualnych osób była realistyczna.**	-2 -1 0 +1 +2
**Ubiór wirtualnych osób był dostosowany do okoliczności.**	-2 -1 0 +1 +2
**Osoby wirtualne różniły się od siebie wyglądem.**	-2 -1 0 +1 +2
**Wirtualne postaci jako grupa wyglądały w tych okolicznościach autentycznie.**	-2 -1 0 +1 +2
**Ubiór wirtualnych osób sprawiał naturalne wrażenie.**	-2 -1 0 +1 +2
**Natężenie dźwięków w przestrzeni wirtualnej odbierałem/łam jako...**	-2 -1 0 +1 +2<br>(zbyt ciche) (idealne) (zbyt głośne)

Polish adaptation of the German VR Simulation Realism Scale (Poeschl & Doering, 2014; on a basis of original, German items)

Klucz:
Realizm sceniczny: 1-5
Realizm zachowania publiczności: 6-9
Realizm wyglądu publiczności: 10-13
Realizm dźwiękowy: 14
