# Supplementary material for: VR Realism Scale—Revalidation of contemporary VR headsets on a Polish sample
Source: PLoS One. 2021 Dec 21;16(12):e0261507. doi: 10.1371/journal.pone.0261507 (PMC8691612; doi:10.1371/journal.pone.0261507)
Supplement: S1 File — (PDF) [file pone.0261507.s002.pdf]

Supporting Information

This document contains supplementary materials for the article 'VR Realism Scale - revalidation of contemporary VR headsets on a Polish sample.'

Natalia Lipp[1], Radosław Sterna[2], Natalia Dużmańska-Misiarczyk[2], Agnieszka Strojny[1], Sandra Poeschl-Guenther[3], Paweł Strojny[1]

[1] Institute of Applied Psychology, Faculty of Management and Social Communication

[2] Doctoral School in the Social Sciences, Jagiellonian University, Krakow, Poland

[3] Research Group for Media Psychology and Media Design, Institute for Media and Communication Science, Ilmenau University of Technology, Germany

Procedures

Study A

The study was an online survey distributed on the Steam platform. The participants were asked to remind themselves about their last interaction with a virtual environment that took place not earlier than seven days ago, during which they controlled a single character in a three-dimensional environment and answer the questions according to their experience and perception of the chosen interaction. They could choose any platform (console, PC, or VR). After providing an informed consent, confirming that they are 18 or more years old, and providing basic sociodemographic information, they were asked several questions about the game/virtual environment they chose:

a) title of the game,
b) what platform did they use,
c) what display did they use,
d) what type of sound was used (mono/stereo/surround/no sound),
e) describe in 1-2 sentences the play session they chose,
f) for how long did they play the game,
g) how long ago did they play the game,
h) what perspective was used in the game (first person/third person from above/third person from behind),
i) were there any other real people in the virtual environment (and did the player interact with them),
j) were there any virtual, non-player characters in the virtual environment (and did the player interact with them),
k) what type of software did they use.

Then, on separate pages, the items of several questionnaires were displayed (see the Measures Section).

Study B

The main purpose of the study was to assess whether there is a difference in physiological arousal and subjectively reported emotions, situational stress, workload, and perception of the virtual reality between a task in a virtual environment (experimental group) and free exploration of that environment (control group). The virtual environment consisted of an accident site on an intersection in a small town with a crashed car and several victims. Some of the possible actions were: conducting a primary interview, checking some physical parameters (breathing, pulse, pain reaction, capillary recurrence, airways), dressing the wounds, covering the victim with a blanket. The actions were chosen using text commands which were visible on the controller. HTC Vive head-mounted display (HMD) and two hand-held controllers were used. The experimental group's task lasted 5 minutes, and it was to perform the Medical Rescue Sequence - a procedure detailed in the National Firefighting Rescue System documentation - on virtual victims present in the virtual environment. The task for the control group was to walk around and explore the virtual environment. No victims were present in the control environment. After the task, the participants completed a set of printed questionnaires (see the Measures section).

Study C

In this study, a between-subject design with four different conditions was used. Firstly, each participant was assigned pseudo-randomly (ensuring equal group sizes) to one of the conditions. Conditions differed in terms of two characteristics, namely task difficulty (easy or difficult) and virtual observers' presence (present or not). Thus, the conditions were as follows:

(1) easy task alone; (2) easy task with observers; (3) difficult task alone; (4) difficult task with observers. All tasks were performed in the virtual reality environment designed in cooperation with developers from Nano Games & Simpro studies. The participants were trained (5 min), and after that, they started the main part of the experiment (5 min). Their task was to move virtual objects (bollards) from one side of the virtual street to another. In the easy condition, the participants performed the task with the controller in their dominant hand, with control buttons working as they were taught in the training session. In the difficult version of the task, they held the controller in their non-dominant hand, with buttons working differently from the training session. The performance measure was the completion time from the start of the task until the last bollard was moved to the other side of the street, measured in seconds.

Study D

The study was the first iteration of a longitudinal study. The main purpose was to assess the influence of several changes in a VR training simulator on participants' subjective feelings and several psychophysiological parameters. The virtual environment consisted of an accident site on an intersection in a small town with a crashed car and several victims - the same as in Study B. There were three experimental groups with different stimuli (i.e., virtual bystanders, a child's toy, a small dog) and one control group. The participants were instructed on how to use the simulator. The available actions and hardware were the same as in Study B, and the task was the same as in the experimental group of Study B. After the task, the participants were asked to complete a set of questionnaires (see the Measures section) on a computer screen. Completing the whole set took approximately 15-20 minutes.

Study E

This study was the second iteration of the longitudinal study described above. The procedure was identical as described in the previous section. There were several changes made to the virtual environment, which aimed to enhance the simulation's realism. Sounds were made adequate in terms of volume (louder) and content. Sounds of people wailing and moaning and car traffic were added. Some models were improved as well - more details were added to the inside of the car, and its doors were made thicker. An animation of opening and closing mouth while checking airways was added. One of the virtual victims would lose consciousness (fall to the ground) 40 s after engaging in an interaction with her. The self-report measures remained the same as in Study D, but this time they were administered using a Python script written in PsychoPy (Peirce, 2007, 2009).

Study F

This study's main purpose was to verify whether virtual stimuli may activate the mortality salience and its psychological consequences (Greenberg, Solomon & Pyszczynski, 1997). 120 students participated in a 2 (death of a virtual agent vs. all agents alive) x 2 (fun game vs. simulator for critical infrastructure operators) factorial between-subject experiment. The virtual environments consisted of either an accident site on an intersection in a small town with a crashed car, several fatal victims and several bystanders (conditions with the death of virtual agents) or the same intersection with several bystanders but without a crashed car (conditions without activation of mortality salience). At the same time, half of the respondents were instructed that this was just a game. The other half were instructed that this was an important simulator designed for emergency services. The current study was crucial for its preparation. The participants were instructed on how to interact with virtual agents and items. Possible actions were as follows: asking agents what happened, asking agents to go away, grabbing and moving items (bollards). The actions with agents were chosen using text

commands which were visible on the controller. HTC Vive head-mounted display (HMD) and two hand-held controllers were used. The task lasted 8 minutes. The task of the participants was to find out what had happened in the virtual environment earlier and to secure the place of the event with bollards. After task completion, participants were asked to fill out several questionnaires.

Measures

The Polish adaptation of the Igroup Presence Questionnaire (IPQ; Schubert, Friedmann & Regenbrecht, 2001) by Strojny, Lipp, and Strojny (unpublished) was used to measure the sense of presence (in three dimensions: spatial presence, involvement, and realness). It consists of 13 items enabling responses on a 5-point Likert scale. The psychometric evaluation of the Polish version revealed satisfying internal consistency coefficients (Cronbach's alpha for three factors > .80) and an accurate model fit.

The Player Need Satisfaction Questionnaire (Ryan, Rigby & Przybylski, 2006) is a questionnaire based on the Self-Determination Theory. It measures the level to which three universal needs (competence, autonomy, and relatedness) are satisfied by playing a game; the questionnaire measures presence/immersion and intuitive controls as well. In the absence of a properly validated Polish version of the questionnaire, we decided to assess the internal consistency of a translation made by the authors of the questionnaire using Cronbach's alpha reliability coefficient. The Cronbach's coefficients were as follows: .79 for competence need, .80 for autonomy need, for .67 relatedness need.

The Immersion Questionnaire (Jennett, Cox, Cairns, Dhoparee & Epps, 2008; Polish adaptation by Strojny & Strojny, 2014) was used to measure the players' absorption in the

virtual environment. It consists of 27 items. Factor analyses performed by its authors confirmed the one-factor structure of the scale.

The Flow State Scale-2 (Jackson & Eklund, 2002; Polish translation/adaptation by Tomczak & Hornowska, 2012) - to assess the experience of flow during a game session. It contains 36 items measuring nine aspects of flow. For the current analysis, the flow variable was calculated as the average result of all items.

The Scale of Aesthetics (Chevalier, Maury & Fouquereau, 2014; Polish adaptation by Strojny & Strojny, 2016) was used to evaluate perceived aesthetical aspects of the graphics' quality (in the classical and expressive dimension). It consists of 10 items enabling responses on a 7-point Likert scale.

The Polish translation of the Co-presence Scale (Poeschl & Doering, 2015) was used for measuring social aspects of VR - particularly, engagement in relationships with virtual characters (in four dimensions: presenter's reaction to virtual agents, perceived virtual agents' reaction, the impression of interaction possibilities and (co-)presence of other people). The items are rated on a 5-point Likert scale. Due to the lack of a properly validated Polish version, the internal consistency of the Polish translation made by the authors of the study was conducted using reliability analysis. The obtained Cronbach's coefficient was high (α = .89).

The Scale of Emotions (Wojciszke and Baryła 2005) was used for assessing the intensity of six basic emotions: joy, love, fear, anger, guilt, and sadness. It consists of 24 items rated on a 5-point Likert Scale.

The Self-Assessment Manikin (SAM; Bradley and Lang 1994) is a pictorial questionnaire. It was used to assess emotional responses to stimuli in three fundamental dimensions: valence, arousal, and dominance.

The Scale of Mood (Wojciszke and Baryła 2005) was used to measure positive and negative moods. It consists of 30 statements that describe the current mood but do not refer to specific emotions. The items are rated on a 5-point Likert scale.

NASA Task Load Index (NASA-TLX, Hart and Staveland 1988, Polish version by Zieliński and Biernacki, 2010) was used to measure subjective task workload. Participants rate their perception of task mental workload, physical workload, time pressure, effort, performance, and frustration.

The General Self-Efficacy Scale (Schwarzer and Jerusalem, 1995; polish adaptation by Juczynski, 2000) was used to measure self-efficacy, which can be defined as the belief in one's competence to cope with stressful demands. In this research program, the scale was used to assess proficiency in the use of a VR simulator. The scale consists of 10 items. Possible responses are *not at all true, hardly true, moderately true, exactly true.*

Stress Appraisal Questionnaire (Włodarczyk and Wrześniewski 2010) was used to measure situational stress (in four dimensions: harm/loss, challenge-activity, challenge-passivity, and threat). The questionnaire is based on Transactional Stress Theory, and it consists of 40 items.

Simulator Sickness Questionnaire (Kennedy, Lane, Berbaum, Lilienthal, 1994) was used to measure the intensity of simulator sickness symptoms (overall and in three dimensions: nausea, oculomotor, and disorientation). The questionnaire consists of 17 items rated from 0 ('*I do not experience this symptom at all*') to 3 ('*I am significantly affected by this symptom*').

Positive and Negative Affect Schedule (Watson, Clark & Tellegen, 1988, polish version by Brzozowski, 2010) was used to measure mood. It was used only in one of our studies (study F) to control the participants' affect during the experimental procedure. The questionnaire consists of 20 items measuring positive and negative affect. These two factors remain strongly uncorrelated, according to Watson and colleagues (1988).

References

Bradley M, Lang P. Measuring emotion: The self-assessment manikin and the semantic differential. Journal of Behavior Therapy and Experimental Psychiatry. 1994;25(1):49-59.

Chevalier A, Maury A, Fouquereau N. The influence of the search complexity and the familiarity with the website on the subjective appraisal of aesthetics, mental effort, and usability. Behaviour & Information Technology. 2013;33(2):117-132.

Greenberg J, Solomon S, Pyszczynski T. Terror Management Theory of Self-Esteem and Cultural Worldviews: Empirical Assessments and Conceptual Refinements. Advances in Experimental Social Psychology. 1997;29:61-139.

Hart S, Staveland L. Development of NASA-TLX (Task Load Index): Results of Empirical and Theoretical Research. Advances in Psychology. 1988;52:139-183.

Jackson S, Eklund R. Assessing Flow in Physical Activity: The Flow State Scale–2 and Dispositional Flow Scale–2. Journal of Sport and Exercise Psychology. 2002;24(2):133-150.

Jennett C, Cox A, Cairns P, Dhoparee S, Epps A, Tijs T et al. Measuring and defining the experience of immersion in games. International Journal of Human-Computer Studies. 2008;66(9):641-661.

Juczyński Z. Poczucie własnej skuteczności–teoria i pomiar. Acta Universitatis Lodziensis. Folia Psychologica. 2000;4:89-94.

Kennedy R, Lane N, Berbaum K, Lilienthal M. Simulator Sickness Questionnaire: An Enhanced Method for Quantifying Simulator Sickness. The International Journal of Aviation Psychology. 1994;3(3):203-220.

Peirce J. PsychoPy—Psychophysics software in Python. Journal of Neuroscience Methods. 2007;162(1-2):8-13.

Poeschl S, Doering N. Measuring co-presence and social presence in virtual environments – psychometric construction of a German scale for fear of public speaking scenario. In Wiederhold BK, Riva G, Wiederhold MD, editors. Annual Review of Cybertherapy and Telemedicine. San Diego: Interactive Media Institute; 2015. pp.58-68.

Ryan R, Rigby C, Przybylski A. The Motivational Pull of Video Games: A Self-Determination Theory Approach. Motivation and Emotion. 2006;30(4):344-360.

Schubert T, Friedmann F, Regenbrecht H. The Experience of Presence: Factor Analytic Insights. Presence: Teleoperators and Virtual Environments. 2001;10(3):266-281.

Schwarzer R, Jerusalem M. Generalized Self-Efficacy Scale. In Weinman J, Wright S, Johnston M, editors. Measures in health psychology: A user's portfolio. Causal and control beliefs. Windsor, England: NFER-NELSON; 1995. pp. 35–37.

Strojny P, Strojny A. Kwestionariusz immersji – polska adaptacja i empiryczna weryfikacja narzędzia [Immersion questionnaire: Polish adaptation and empirical verification.]. Homo Ludens. 2014; 1(6): 187–198.

Strojny P, Strojny A. Czy piękno gry ma znaczenie? Związek estetyki klasycznej i ekspresyjnej z zaangażowaniem w grę wideo. [Does the beauty of the video game matter? Relationship between aesthetics and engagement in playing]. Annales Universitatis Paedagogicae Cracoviensis. Studia Psychologica. 2016; 9: 35–41.

Tomczak M, Hornowska E. Stan optymalnego zaangażowania (flow) a style radzenia sobie w sytuacjach stresowych u osób uprawiających sport. Wychowanie Fizyczne i Sport. 2012; 2: 5–31.

Watson D, Clark L, Tellegen A. Development and validation of brief measures of positive and negative affect: The PANAS scales. Journal of Personality and Social Psychology. 1988;54(6):1063-1070.

Włodarczyk D, Wrześniewski K. Kwestionariusz Oceny Stresu (KOS). [The Questionnaire of Stress Appraisal]. Przegląd Psychologiczny, 2010; 53: 479–496.

Wojciszke B, Baryła W. Skale do pomiaru nastroju i sześciu emocji. [Scales measuring mood and six emotions]. Czasopismo Psychologiczne. 2005; 11(1):31–48.

Zieliński P, Biernacki M. Analiza psychometryczna polskiego przekładu narzędzia do subiektywnej oceny obciążenia zadaniowego NASA-TLX. Polski Przegląd Medycyny Lotniczej. 2010; 3:219–239.
