# Supplementary material for: VR Realism Scale—Revalidation of contemporary VR headsets on a Polish sample
Source: PLoS One. 2021 Dec 21;16(12):e0261507. doi: 10.1371/journal.pone.0261507 (PMC8691612; doi:10.1371/journal.pone.0261507)
Supplement: Attachment — Submitted filename: Response to Reviewers.docx [file pone.0261507.s005.docx]

**Authors' responses to the Reviewers' comments**

First of all, we would like to thank the Reviewers for rereading the manuscript. We used their advice and improved our text. The detailed answered are provided below.

# Journal Requirements

1. **Please review your reference list to ensure that it is complete and correct. If you have cited papers that have been retracted, please include the rationale for doing so in the manuscript text, or remove these references and replace them with relevant current references. Any changes to the reference list should be mentioned in the rebuttal letter that accompanies your revised manuscript. If you need to cite a retracted article, indicate the article's retracted status in the References list and also include a citation and full reference for the retraction notice.**

We reviewed our reference list – there are no retracted positions. However, due to changes made to the text, the order of position has been changed.

# Reviewer 1

1. **Both in CFA and Bifactor model, the authors used the fixed load method (e.g., the factor loading for the first item of per factor is fixed to 1), but I'm a little unsure that the standardized factor loading of the first item is also 1, please check it carefully.**

Thank you for your vigilance. You were right from the beginning – standardized factor loadings are not fixed to 1, so we've corrected the figures.

# Reviewer 2

1. **There is only one minor concern: could you please reorganize your introduction session to make it more concise, it is quite long and hard o get the main purpose/ point. For example, you can briefly interpret why you consider "classical aesthetics" as the mediator in introduction session, and then add the "hypotheses session" to provide more arguments for that.**

Thank you for this remark. We followed your suggestion and provided arguments about aesthetics in the '*Aims of our study*' section next to other hypotheses.

In addition to your comment about reorganizing the introduction, you pointed out that we don't share data and the language errors in the text. We have completed the dataset with the data (to calculate measurement invariance) which we received from the German team. These data can be found in the spreadsheet named 'CFA+item_analysis_invariance.' Additionally, a professional English language editor proofread the text, correcting all errors.
